# Crush Injury to the Hand Following Epileptic Seizure Leading to Compartment Syndrome

**DOI:** 10.29252/wjps.7.3.364

**Published:** 2018-09

**Authors:** Amitabh Thacoor, Saif Ramman, Norbert Kang

**Affiliations:** Department of Plastic and Reconstructive Surgery, Royal Free Hospital, Pond Street, London, UK

**Keywords:** Compartment syndrome, Hand, Plastic surgery, Epilepsy

## Abstract

Compartment syndrome of the forearm or leg has been well documented in the literature. However, there have been few published reports of hand compartment syndrome. We hereby present the first reported case in the literature of hand compartment syndrome secondary to an epileptic seizure. A 50-year old gentleman with known epilepsy presented to the Emergency Department following a witnessed tonic-clonic seizure. The patient’s chief complaints were a grossly swollen and excruciatingly painful dominant right hand. Examination revealed severely reduced range of motion and neurovascular compromise. An emergency decompression fasciotomy was performed in the operating theatre, where severe oedema was noted with viable muscle throughout. Compartment syndrome can occur in any muscle compartment of the body, including in the hand. Any crush injury to the hand should trigger a high index of suspicion by the clinician to enable prompt recognition of this surgical emergency and initiate timely management.

## INTRODUCTION

Compartment syndrome is a group of symptoms arising from an increase in interstitial pressure within a closed confined fascial space, ultimately resulting in vascular compromise to the compartment tissue. Compartment syndrome of the forearm and leg has been well described as the most common areas to be affected. However, a compartment syndrome can develop in any compartment made of muscle enclosed in fascia, such as the hand. We present the case of hand compartment syndrome in a patient following an epileptic seizure.

## CASE REPORT

**Fig. 1:**
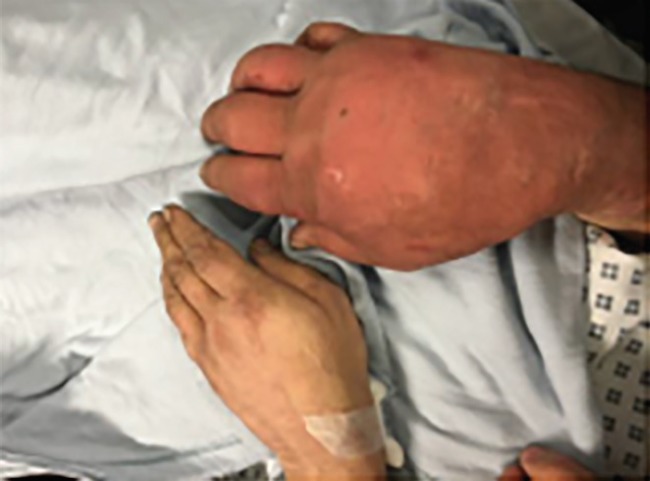
Pre-operative photo showing clear swelling of the right hand

A 50-year old homeless gentleman, with a background of known epilepsy and chronic obstructive pulmonary disease, presented to the Emergency Department following a fall following a 7-minute witnessed tonic-clonic seizure. He complained of a rapidly worsening, excruciating pain generally all over his dominant right hand since the fall. Immediate referral to our plastic surgery team was performed. On examination, there was obvious bruising and swelling extending to the mid-forearm, with significantly reduced range of motion at the wrist and all finger joints as well as reduced sensation generally in the affected hand, particularly in the median nerve distribution (Figure 1). 

**Fig. 2:**
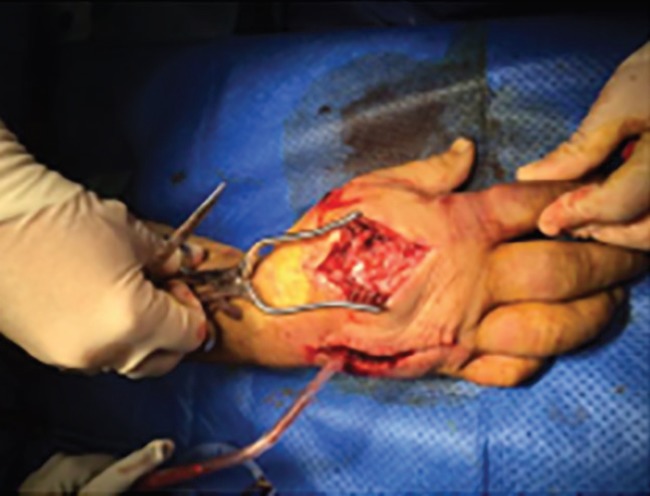
Longitudinal fasciotomy to decompress dorsal interosseous muscles

**Fig. 3:**
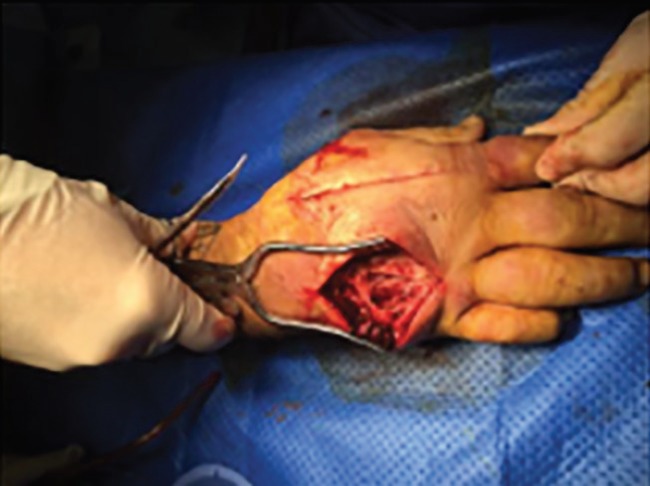
Longitudinal fasciotomy to decompress dorsal interosseous muscles

**Fig. 4:**
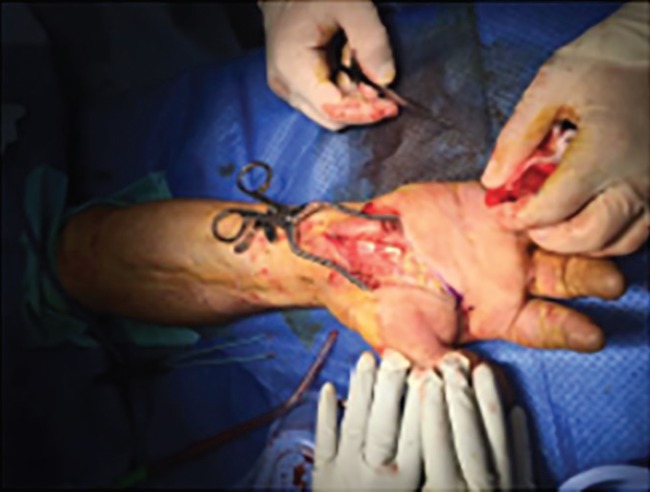
Decompression of carpal tunnel and hypothenar eminence

The radial and ulnar pulses were not palpable. Following an unremarkable hand radiograph, a diagnosis of acute hand compartment syndrome secondary to a crush injury was established, and immediate surgical exploration was performed 8 hours after the injury to decompress all dorsal and volar compartments, thenar and hypothenar compartments and mid palmar space (Figures 2-4). Although significant oedema was noted above and below the deep fascia, muscle was viable throughout. All incisions were left open and the hand dressed and immobilised in a volar splint. Strict post-operative elevation in a Bradford sling followed on the ward and the patient later went home. 

## DISCUSSION

Compartment syndrome can present with various aetiologies, often of traumatic origin such as fractures, crush injuries, compressive dressings, burns, fluid extravasations or bites. If untreated, compartment syndrome can have permanent, drastic consequences. As intercompartmental pressure rises, capillary perfusion pressure is exceeded, resulting in hypoperfusion of muscle tissues, jeopardising tissue oxygenation and cellular viability. This precipitates local hypoxia, neurological dysfunction and eventually muscle necrosis with release of systemic toxins, fibrosis and contractures. 

These were first described by Richard von Volkmann in 1881, whereby he noted irreversible hand flexor muscle contractures secondary to an ischaemic injury. In addition, nerve injury leads to further muscle dysfunction, sensory impairments and chronic pain. The clinical picture of acute compartment syndrome is an evolving one, which can make diagnosis particularly difficult. Classically, pain out of proportion to the underlying nature of the injury is described in a tense, swollen hand. This pain is often progressive and persistent and increases with passive motion of the muscles in that compartment. Later signs include pulselessness and skin pallor, which both indicate a degree of arterial insufficiency, and paraesthesia and paralysis, both suggesting neurological impairment. 

The hand is made up of eleven distinct compartments in most cases. These include three volar interossei compartments, four dorsal interossei compartments, a thenar compartment, a hypothenar compartment, an adductor compartment and a midpalmar compartment.^,^ The blood supply to these compartments arises from the deep and superficial arches which originate from the radial and ulnar arteries. Compartment syndrome diagnosis is a clinical one. Signs and symptoms outlined above should raise the suspicion of hand compartment syndrome in the early onset. However, in equivocal cases and the absence of a diagnosis, measuring intracompartmental pressures can be performed. This can be of particular value in the intoxicated or uncooperative patient. 

Much debate exists regarding what compartment pressure should indicate a fasciotomy. Currently, guidelines recommend a fasciotomy in cases where intracompartmental pressures are within 30mm Hg of the diastolic pressure, within 45 mmHg of the mean arterial pressure or greater than 30 mmHg 3.^10^ It is however advocated that despite measurements in intracompartmental pressures, the surgeon should not hesitate to open a compartment which looks suspicious.^,^ The definitive treatment for compartment syndrome following diagnosis is urgent fasciotomy and decompression of the affected compartments. Classically, decompression should include two longitudinal dorsal incisions over the second and fourth dorsal compartments, a thenar eminence incision and a carpal tunnel release 6. 

This was the approach chosen in our patient. Bulging muscles confirm the diagnosis. Closure, either primarily or with split skin grafting can then be performed at a later date. Compartment syndrome is a well-recognised surgical emergency. Clinical diagnosis can be challenging, particularly in the early stages of its clinical course. Although the forearm and the legs are the most common areas to be affected, compartment syndrome can present in any muscle compartment of the body, particularly in the hand.

A high index of suspicion is required and careful monitoring of evolving nature of the pain is paramount. Swelling and worsening pain exacerbated by passive stretch of the muscles are the characteristic features of compartment syndrome affecting the hand. Pulselessness and parasthesia are often absent. Despite the use of intracompartmental pressure measurements, clinical examination remains the most crucial deciding factor for emergency fasciotomy. Any crush injury to the hand resulting in swelling should raise suspicions from the clinician and failure to act promptly can result in drastic consequences such as tissue necrosis, neurological sequelae and ultimately amputation.

## CONFLICT OF INTEREST

The authors declare no conflict of interest.
